# Juvenile idiopathic arthritis-associated uveitis

**DOI:** 10.1186/s12969-016-0088-2

**Published:** 2016-04-27

**Authors:** Sarah L. N. Clarke, Ethan S. Sen, Athimalaipet V. Ramanan

**Affiliations:** Department of Paediatric Rheumatology, Bristol Royal Hospital for Children, Upper Maudlin Street, Bristol, BS2 8BJ UK; School of Clinical Sciences, University of Bristol, Bristol, UK

**Keywords:** Juvenile idiopathic arthritis, Uveitis, Epidemiology, Pathogenesis, Screening, Biologics, Prognosis

## Abstract

Juvenile idiopathic arthritis (JIA) is the most common rheumatic disease of childhood, with JIA-associated uveitis its most common extra-articular manifestation. JIA-associated uveitis is a potentially sight-threatening condition and thus carries a considerable risk of morbidity. The aetiology of the condition is autoimmune in nature with the predominant involvement of CD4^+^ T cells. However, the underlying pathogenic mechanisms remain unclear, particularly regarding interplay between genetic and environmental factors. JIA-associated uveitis comes in several forms, but the most common presentation is of the chronic anterior uveitis type. This condition is usually asymptomatic and thus screening for JIA-associated uveitis in at-risk patients is paramount. Early detection and treatment aims to stop inflammation and prevent the development of complications leading to visual loss, which can occur due to both active disease and burden of disease treatment. Visually disabling complications of JIA-associated uveitis include cataracts, glaucoma, band keratopathy and macular oedema. There is a growing body of evidence for the early introduction of systemic immunosuppressive therapies in order to reduce topical and systemic glucocorticoid use. This includes more traditional treatments, such as methotrexate, as well as newer biological therapies. This review highlights the epidemiology of JIA-associated uveitis, the underlying pathogenesis and how affected patients may present. The current guidelines and criteria for screening, diagnosis and monitoring are discussed along with approaches to management.

## Background

JIA is the most common rheumatic disease of childhood, with JIA-associated uveitis (JIA-U) its most frequent extra-articular manifestation. Uveitis is the inflammation of the uvea (comprising the iris, choroid and retina). The Standardisation of Uveitis Nomenclature (SUN) criteria are used to define the anatomical location and time course of uveitis, allowing reproducible assessment and monitoring of disease [1, 2]. The time course of uveitis is defined as acute, subacute, chronic or recurrent and anatomical location as anterior, intermediate, posterior or panuveitis. Additionally, JIA-U can be unilateral or bilateral, thus assessments are made of each eye in turn. JIA-U most commonly presents as chronic anterior uveitis, which is often clinically silent. This form of uveitis is most frequently associated with oligoarticular and rheumatoid factor negative polyarticular categories of JIA. In contrast, acute anterior uveitis, which is generally symptomatic, unilateral and episodic, is seen particularly in the enthesitis-related arthritis (ERA) category of JIA. Early identification and treatment of JIA-U is important given the risk of sight-threatening complications. Management of JIA-U includes the use of both topical and systemic agents and is an active area of research. Visually-disabling complications can occur both as a result of chronic disease activity and treatment burden (particularly topical glucocorticoids), and include cataracts, glaucoma, band keratopathy and macular oedema [3].

## Epidemiology

Uveitis is predominantly a disease of adults, with children representing approximately 5–10 % [4]. However, the risk of delayed diagnosis and onset of complications at a young age makes this an important clinical problem. The overall incidence of uveitis in the paediatric population, reported in a study from Finland, was 4.3 per 100,000/year and prevalence of 27.9 per 100,000 [5]. When broken down by aetiology, the prevalence of JIA-U among all causes of paediatric uveitis varies widely by referral centre, ranging from 15–67 % across centres in Europe, North America and Israel [4, 6–9]. The variation in these figures may partly be due to the referral cohorts from which the patients are selected, however, it should also be noted that uveitis can precede a diagnosis of arthritis in 3–7 % of children with JIA [10] and thus children presenting with uveitis need careful assessment for underlying systemic or infectious disease. When looking specifically at the prevalence of uveitis in those patients already known to have JIA, estimates of prevalence ranges from 11.6 % [11] to 30 % [12] although overall it appears to be decreasing over the past decade. Thus there is a well-established, reciprocal link between JIA and uveitis; uveitis is a frequent finding in JIA patients as well as JIA being a common underlying cause for the condition in children.

Regarding disease pattern, in one study where 13.1 % of 1081 JIA patients developed uveitis, chronic anterior uveitis was predominant (68.3 %) [13]. However, acute anterior disease (16.2 %), recurrent anterior disease (12 %) and panuveitis (3.5 %) were also encountered.

A number of risk factors for JIA-U have been identified. These include gender, JIA category, age of onset, and ANA and HLA-B27 positivity [3, 10, 12, 14]. A younger age, female gender, oligoarticular disease and presence of ANA are risk factors for chronic anterior uveitis. In contrast, being male with HLA-B27 and ERA predispose to acute anterior uveitis. The interplay between individual risk factors is likely to be complex and inter-dependent. For example, in a retrospective study of 1047 patients with JIA, the risk of developing uveitis was age-dependent in girls, but not boys [15]. The impact of ethnicity on JIA-U is unclear. Early studies identified JIA-U in many different ethnic groups and suggested ethnicity did not play a role [16]. More recent studies have suggested that those of European descent are at increased risk of JIA-U compared to the at-risk local population [17].

## Pathogenesis

Despite the well-documented link between JIA and uveitis, the reason for uveal inflammation is not well understood. It is likely that the pathophysiology of JIA-U involves both genetic and environmental elements. The genetic basis for JIA-U is likely to be complex; despite familial cases having been reported [18], monogenic or Mendelian patterns of inheritance have not been identified [19].

Most JIA-associated genes lie within the human leucocyte antigen (HLA) region, and this association supports the theory of JIA-U being an autoimmune disorder. Studies looking at the association between risk of JIA-U and HLA subtype have conflicted, however HLA alleles which confer risk have been identified when looking at HLA alleles within particular JIA categories. In patients with oligoarticular JIA, chronic anterior uveitis has been associated with the HLA-DR5 haplotype [20] and HLA-DRB1*1104 allele [21]. In particular the combination of HLA-DRB1*1104 and HLA-DPB1*0201 alleles is linked with a 7.7-fold increased risk of chronic uveitis. HLA-B27 is classically seen in ERA and confers an increased risk of acute anterior uveitis in this patient group [22]. HLA-DR1 is the only HLA allele which has repeatedly been shown to be protective against chronic anterior uveitis associated with JIA [23]. Additionally, there is evidence that HLA associations are temporal with HLA alleles conferring protection or susceptibility to JIA at different ages; some alleles confer protection in early children but increase risk of JIA-associated uveitis in later childhood [24].

At the cellular level, there appears to be involvement of both T and B lymphocytes in generating an immune response against native intraocular antigens including S-arrestin (also known as retinal S-antigen), retinol-binding protein 3, and tyrosinase-related proteins [25]. Evidence for the involvement of both B and T lymphocytes comes from immunohistochemistry of eye biopsies from patients with JIA which show a predominance of CD4^+^ rather than CD8^+^ T lymphocytes as well as variable levels of CD20^+^ B lymphocytes. CD4^+^ lymphocytes include pro-inflammatory Th1 cells (producing interferon gamma) and Th17 cells (producing interleukin-17), which are regulated by both CD4^+^CD25^+^FoxP3^+^T regulatory cells (Tregs) and inducible Tregs. It is likely that autoimmunity results from imbalance between these cell subsets leading to loss of tolerance to self-antigens. In addition to the adaptive immune response, elements of the innate immune system have also been implicated in the pathogenesis of JIA-U [23].

The risk of chronic anterior uveitis in JIA patients is increased in those who are also ANA positive [26], raising the question as to whether ANAs are pathogenic in, or an epiphenomenon of, JIA-U. The specificity of the ANAs is not known although studies have previously shown higher frequency of anti-histone antibodies in JIA patients with uveitis compared to those with no historic or current uveitis [27, 28]. Another report, however, has not shown temporal association between anti-histone antibodies and the presence of uveitis [29]. Studies have been undertaken to address the role of ANAs by using immunofluorescence to detect antibody binding in human eyes incubated with the sera from JIA patients versus sera from healthy controls. JIA patients were shown to have increased frequency of antibodies against the iris and retina [30]. Similar findings were seen when looking at antibody binding using JIA patient (with or without uveitis) and healthy control sera in swine eyes. Walscheid et al. showed predominant binding of antibody from JIA-U patients to the iris and ciliary body [31]. Whether these antibodies are a cause or an effect of uveitis has not been elucidated by these studies, as they used sera from patients with established disease. To date, no specific intra-ocular antigen has been identified as the target for ANAs, and thus their role in pathogenesis remains unclear.

## Clinical features

Uveitis can present with overt symptoms as seen in acute anterior uveitis. Typical features include eye pain, redness, headaches, photophobia and visual changes. However, chronic anterior uveitis, more commonly seen in JIA, is often completely asymptomatic. Hence, regular screening for uveitis in JIA patients is essential to detect clinically-silent, but potentially vision-threatening, disease. Even in the presence of symptoms, children may be unable to report reliably what they are experiencing, thus a need for formal assessment of vision and ocular health remains.

Sabri et al. found that the mean time from onset of JIA to onset of uveitis was 1.8 years [13]. However, uveitis can pre-date the diagnosis of JIA thus eye inflammation can go unnoticed for a significant period. Additionally, a recent study has suggested a biphasic course for the condition with a second peak of disease activity occurring around puberty [32] suggesting the need for vigilance in monitoring these patients over time.

## Diagnosis/Screening

All patients at risk of JIA-U should be screened for the condition. Screening guidelines are available in several countries including the UK [33]. The UK guidelines are summarised in Box 1. The UK consensus standard is for first ophthalmological assessment to take place within 6 weeks of JIA being diagnosed or suspected [34], underlining the importance of diagnosing and treating this condition promptly.

Box 1: British Society for Paediatric and Adolescent Rheumatology/ Royal College of Ophthalmology guidelines for uveitis screening in JIA [33].

Screening of children with JIA for uveitis involves a combination of slit lamp examination, measurement of intraocular pressure and age-appropriate visual acuity (VA) testing. A slit lamp allows examination of the anterior and posterior chambers as well as the retina. A diagnosis of uveitis is made based on features of inflammation on slit lamp examination. These include cells in the anterior chamber (AC) [35] and AC flare resulting from protein leakage into the AC due to breakdown of the blood–aqueous humour barrier [36]. The SUN criteria provide a grading system for intra-ocular inflammation, which takes into account AC cells, AC flare, vitreous cells, and vitreous haze or debris (Box 2). The criteria also provide definitions of improvement and worsening of the condition (Box 2) allowing reproducible assessment and monitoring of uveitis activity.

Box 2: Standardisation of Uveitis Nomenclature (SUN) criteria for uveitis activity [adapted with permission from Elsevier © Jabs et al. Am J Ophthalmol 140, 509-16 (2005)[2]]

The measurement of intra-ocular pressure is important in patients with JIA-U as patients are at risk of intraocular hypertension and glaucoma. This risk remains, despite control of active inflammation, as illustrated by one study showing the first measurement of raised intraocular pressure at a time when the disease was inactive in 60 % of eyes [37]. Hence the need for regular measurement of intra-ocular pressure during follow-up.

The assessment of VA provides a measure of both disease activity and visual damage resulting from both chronic disease activity and failure or complications of treatment. There are a number of structural complications which occur in the setting of JIA-U which contribute to visual loss. These include band keratopathy, posterior synechiae, cataract, glaucoma, hypotony, macular oedema, epiretinal membrane, and optic disc oedema. The need for functional, age-appropriate assessment of vision during uveitis screening has been highlighted by Heiligenhaus et al. who have developed guidelines for measuring outcome in JIA-U and included assessment of VA as a key outcome measure [38].

## Treatment

**Fig. 1:**
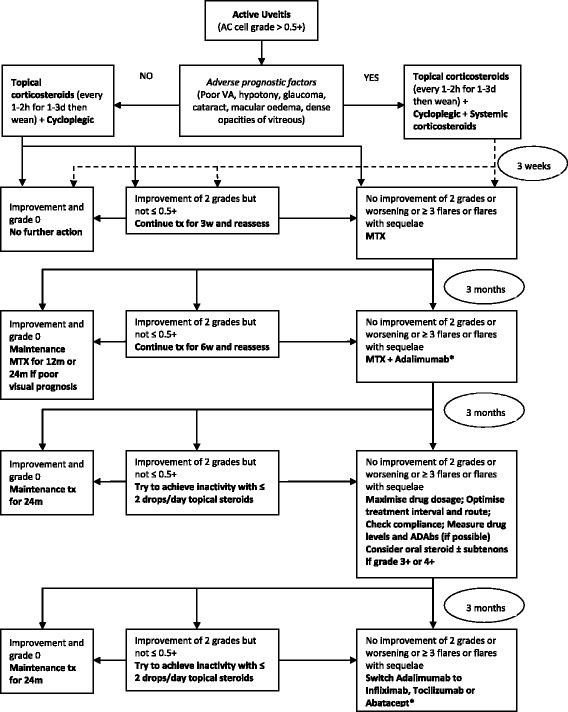
Treatment algorithm for chronic anterior uveitis associated with juvenile idiopathic arthritis. [Adapted with permission from Springer © Bou, R. et al. *Rheumatol Int* 35, 777–785 (2015) [36] and from Springer © Heiligenhaus, A. et al. *Rheumatol Int* 32, 1121–1133 (2012) [38]]. At all stages aim to minimise topical steroid to ≤ 2 drops/day while maintaining AC cell grade ≤ 0.5+. * Mycophenolate mofetil (MMF) is a potential alternative to a biologic drug if there is active uveitis but no active arthritis. Legend: AC: anterior chamber, d: days, h: hours, m: months, MTX: methotrexate, po: by mouth, sc: subcutaneous, tx: treatment, VA: visual acuity, w: weeks

Central to the management of JIA-U is its early detection and appropriate assessment of disease activity. This requires effective communication between paediatric rheumatologists and ophthalmologists which may be helped by organisation of multi-disciplinary clinics. The aim of treatment is to achieve 0 cells in the anterior chamber (SUN AC cell grade 0) in both eyes [39]. Practical management protocols have recently been published both by our group in Bristol, UK [40] and an interdisciplinary panel from Spain [39]. A modified algorithm based on consensus guidelines [39–41] is provided in Fig. 1. Topical glucocorticoids are used in the management of acute anterior uveitis but all other treatments discussed below are in relation to chronic anterior uveitis.

Interdisciplinary guidelines on management of JIA-U advise that therapy is initiated when the AC cell grade is > 0.5+ [41]. Treatment is also indicated when there is fibrin in the AC and keratocytic precipitates with corneal oedema and loss of VA. Immunosuppressive treatment should be intensified if there is failure to see improvement in inflammation or presence of poor prognostic factors. Heiligenhaus et al. identify these factors as poor initial vision, cataract, macular oedema, dense vitreous body opacification, ocular hypotony and glaucoma. Cataract, glaucoma, synechiae and band keratopathy alone, in the absence of active uveitis, do not require anti-inflammatory treatment [41].

### Topical glucocorticoids

The first line treatment for both acute and chronic anterior uveitis is topical glucocorticoids [41–43]. The greatest efficacy is seen with high potency steroids such as prednisolone acetate 1 % or dexamethasone phosphate 0.1 % used once daily to hourly depending on the degree of inflammation [44]. Persistent treatment with frequent steroid eye drops is associated with higher incidence of cataracts. In an observational study, treatment of ≤ 3 drops daily of topical glucocorticoid was associated with an 87 % lower risk of cataracts than > 3 drops daily (RR 0.13, 95 % CI 0.02–0.69; *p* = 0.02) [45].

### Cycloplegics

Cycloplegics are used topically to prevent formation of synechiae by dilating the pupil in patients without synechiae or with grades 1–2 (up to 180°). Options include tropicamide or cyclopentolate 0.5–1 % eye drops [39, 40].

### Systemic and regional glucocorticoids

In severe or sight-threatening JIA-U, where rapid control of intraocular inflammation is required, systemic glucocorticoids either orally (prednisolone 1–2 mg/kg/day) or as intravenous pulse (methylprednisolone 20–30 mg/kg/day for 1–3 days) are sometimes necessary [43]. Evidence for their use derives only from adult studies [46, 47], although they are listed as options in paediatric guidelines [41]. Periocular or intraocular glucocorticoids are occasionally used for severe uveitis. The well-known side effects of systemic glucocorticoids mandate weaning as soon as possible with earlier introduction of steroid-sparing immunosuppression in moderate to severe JIA-U [48].

### Synthetic DMARDs

The primary indication for systemic immunosuppression with one of the DMARDs is failure of adequate control of inflammation after 3 months of topical treatment, particularly with > 3 drops daily [41]. Recurrence of disease when topical glucocorticoids are weaned is also an indication for systemic therapy. Table 1 summarises the range of non-biological immunosuppressants used to treat JIA-U, their doses and evidence base. Evidence comes predominantly from retrospective case series since controlled clinical trials of the drugs in JIA-U have not been undertaken.

**Table 1:** Synthetic DMARDs used in treatment of chronic anterior uveitis associated with JIA

Drug name	Mechanism	Dosage and route	Common side effects	Evidence	Key references
Methotrexate	Cellular adenosine release [95]	10–15 mg/m^2^(or 0.3–0.6 mg/kg) po or sc once weekly	GI discomfort, nausea, elevated liver enzymes	Systematic review and meta-analysis of retrospective case series (*n* = 135): improvement in 73 %	[49]
Azathioprine	Purine nucleoside analogue, inhibits DNA replication	1 mg/kg od, increasing to maximum 3 mg/kg od	GI discomfort, bone marrow suppression, liver impairment	Retrospective case series (*n* = 41): uveitis inactivity in 61.5 % as initial monotherapy; 66.7 % as combination therapy	[96]
Mycophenolate mofetil	Inhibitor of inosine-5-monophosphate dehydrogenase	300 mg/m^2^ bd, increasing to 600 mg/m^2^ bd	GI discomfort, leukopenia, hair loss	Several retrospective case series (*n* = 17, 52 and 85; not all with JIA, variable outcome measures): response in 55–88 %	[97–99]
Ciclosporin	Calcineurin inhibitor blocking T cell proliferation	2.5–5 mg/kg/day in 2 doses	GI disturbance, hypertension, renal and liver dysfunction, lipid abnormalities	Retrospective case series (*n* = 82 and 14): uveitis inactivity in 24 % as monotherapy, 48.6 % as combination therapy	[100, 101]
Tacrolimus	Calcineurin inhibitor blocking T cell proliferation	50–150 microgram/kg bd	GI disturbance, hypertension, renal and liver dysfunction, lipid abnormalities, blood disorders	Retrospective case series (*n* = 62, mostly adults with idiopathic uveitis): permitted glucocorticoid tapering and improved visual acuity	[102]

*Legend*: *bd* twice daily, *GI* gastro-intestinal, *od* once daily, *po* by mouth, *sc* subcutaneous

Methotrexate (MTX) remains the first second-line therapy after topical glucocorticoids. It is indicated after 12 weeks of topical treatment if there is no improvement to AC cell grade ≤ 0.5+, or sooner if > 2 drops daily are required, if there is worsening inflammation or if ocular complications develop [39, 41]. A systematic review and meta-analysis identified 9 eligible studies of methotrexate in non-infectious uveitis including 135 patients of whom 121 had JIA [49]. The dose of 15 mg/m^2^ once weekly was most commonly used, with a maximum of 20 mg orally or 25 mg by subcutaneous injection [50]. The mean time to MTX-induced remission was reported as 4.25 months with duration of remission of 10.3 months [50]. In the systematic review, improvements in intraocular inflammation were seen in 73 % (95 % CI 67–81 %). Adverse events, most commonly gastrointestinal discomfort, nausea and elevated liver enzymes, were experienced in 19.6 % of patients where data were available [49]. MTX treatment was associated with a reduced need for cataract extraction, required in 29 % of treated patients compared with 64 % of those never receiving MTX [51].

One study has reported on uveitis relapse after withdrawal of MTX. Among 22 patients with JIA-U who were treated with MTX, the drug was discontinued in 59 % because of inactive disease after a mean of 1.5 years of inactivity and a total 3.1 years duration of therapy [52]. Factors associated with significantly longer relapse-free survival were: treatment > 3 years, age > 8 years when MTX was withdrawn, and inactivity of uveitis for longer than 2 years before withdrawal. One year increase in duration of inactive uveitis before withdrawal of MTX was associated with a decreased hazard of relapse of 93 %. It is recommended that MTX is continued for at least 12 months once uveitis is inactive and for 24 months in those with poor visual prognosis [39].

Other DMARDs such as mycophenolate mofetil (MMF), tacrolimus, azathioprine, and ciclosporin are used less frequently in treatment of JIA-U [43, 53, 54]. Their use is detailed in Table 1. Leflunomide, which is occasionally used to treat joint disease in JIA, has been associated with more frequent uveitis flares compared with MTX in one retrospective study [55]. Combinations of two DMARDs (such as MTX and MMF) have been tried in more resistant cases. However, we have previously reported that addition of a third immunosuppressive agent has limited efficacy and is associated with increased risk of infections [56]. Current treatment algorithms recommend that if there is worsening disease or failure to achieve AC cell grade 0 after 3–4 months on MTX, then a biologic drug is added [39, 57].

### Biologic drugs

Over the past decade, randomised controlled trials (RCTs) of biologic agents have demonstrated their efficacy in controlling joint disease in JIA [58]. The same drugs have also been used in treatment of associated uveitis (Table 2). The greatest evidence, thus far derived from cohort studies and with RCTs underway, supports the use of adalimumab in treatment of JIA-U. A double-blind, placebo-controlled RCT of adalimumab was stopped early due to efficacy after randomising 90 patients [59]. Analysis of the primary endpoint (“time to treatment failure”) showed a positive effect in favour of adalimumab with a hazard ratio of 0.27 (95 % CI 0.13–0.52, *p* < 0.0001). Adverse events were noted in 88.3 % of patients on adalimumab and 90 % on placebo, with infections being the most common serious adverse event in the treatment group.

**Table 2:** Biological immunosuppressants used in treatment of chronic anterior uveitis associated with JIA

Target	Drug name	Drug class	Dosage and route	Evidence	Key references
TNFα	Etanercept	Dimeric fusion protein	Not recommended for treatment of JIA-U	RCT: no more effective than placebo. Case reports of new uveitis on etanercept	[60, 61]
	Infliximab	Chimeric (mouse-human) mAb	6 mg/kg IV initially, then 3–10 mg/kg. 2^nd^ dose at 2 weeks, then every 4–8 weeks depending on response	Several case series showing efficacy	[61]
	Adalimumab	Fully human mAb	24 mg/m^2^ sc q2w	Several case series showing efficacy. RCTs in progress	[61, 90]
			In practice often 20 mg sc q2w (body weight <30 kg), 40 mg sc q2w (body weight ≥30 kg)		
	Golimumab	Fully human mAb	50 mg sc q4w	Case series (*n* = 3) showing efficacy	[103]
IL-6	Tocilizumab	Humanised mAb	10 mg/kg (body weight <30 kg), 8 mg/kg (body weight >30 kg) IV q4w	Case series (*n* = 3) and case report showing efficacy. Phase II trial in progress	[92, 93, 104, 105]
CD80/86 (CTLA4)	Abatacept	Fully human fusion protein	10 mg/kg IV at weeks 0, 2, 4 then q4w	Case series (*n* = 7 and *n* = 2) showing efficacy. Lack of sustained response in severe uveitis (*n* = 21)	[94, 106–108]
CD20	Rituximab	Chimeric (mouse-human) mAb	375 mg/m^2^ or 750 mg/m^2^ IV, two doses 2 weeks apart	Case series (*n* = 10 and *n* = 8 with long-term follow-up) showing efficacy in most patients	[109–111]

*Legend*: *CTLA-4* cytotoxic T-lymphocyte-associated antigen 4, *IL* interleukin, *IV* intravenous, *JIA-U* juvenile idiopathic arthritis-associated uveitis, *mAb* monoclonal antibody, *od* once daily, *ow* once per week, *q2w* every 2 weeks, *q4w* every 4 weeks, *RCT* randomised controlled trial, *sc* subcutaneous, *TNF* tumour necrosis factor

In contrast to adalimumab, a double-blind RCT of etanercept in 12 patients with JIA-U showed no difference between the drug and placebo [60]. With this small number of patients, the study was powered to detect a difference only if greater than 70 percentage points between treatment arms. Etanercept is not recommended in patients with JIA-U. A meta-analysis including 229 children with JIA-U has shown that infliximab and adalimumab have similar efficacy and both are superior to etanercept [61]. However, during 40 months’ follow-up, uveitis more commonly remained in remission in those treated with adalimumab compared with infliximab (60 % vs 18.8 % respectively) [62]. A small case series has reported that switching between anti-TNF agents, particularly from infliximab to adalimumab, can regain control of uveitis [63]. There have been no systematic studies of treatment options after failure of an anti-TNF although other biologics are sometimes used. Experience using abatacept, tocilizumab and rituximab for treatment of JIA-U has been reported in case series (Table 2). Efficacy was seen in most patients although only small numbers have been reported.

The duration of maintenance therapy on biologic agents is not certain although consensus recommendations suggest continuing treatment for 24 months of inactive disease [39]. One retrospective cohort study (*n* = 50, 44 % with JIA) has reported on uveitis reactivation after stopping infliximab (*n* = 45) or adalimumab (*n* = 5) [64]. Of 19 patients who achieved remission and were subsequently withdrawn from anti-TNFs, 63.8 % had reactivation within 12 months and there did not appear to be an association with duration of medication-induced remission.

There are increasing data on the safety of biologic drugs in JIA and rates of adverse events such as uveitis. Several studies have reported flares or new-onset uveitis while on etanercept [65–67]. Evidence from national registries suggests that etanercept is associated with a greater number of uveitis cases than adalimumab or infliximab [68]. However, no definite causative effect of etanercept can be proved from these retrospective observational studies and the prescribing pattern of the different anti-TNFs may be a confounding factor [69]. A German registry study (*n* = 3467 patients) suggested that in those patients with a known negative past history of uveitis the rate of a new uveitis event was 3.2/1000 patient years (PY) in the MTX group, 1.9/1000PY in the etanercept monotherapy group and 0.9/1000PY in the group on the combination of both [70]. An observational study reporting adverse events in JIA patients receiving biologics in Finland (*n* = 348 patients) reported a rate of new-onset uveitis of 0.8/100PY, 0.3/100PY and 0.5/100PY while on etanercept, infliximab and adalimumab respectively [71]. The rates of uveitis flare were 2.8/100PY, 8.0/100PY and 3.8/100PY for each respective treatment. The authors suggest that the apparently higher rate of flare while on infliximab is because most patients with a pre-existing diagnosis of uveitis were started on this drug during the observation period (1999–2009).

### Surgical treatments

Several complications of uveitis, such as cataracts and glaucoma, may require surgical treatment. Removal of the lens by phacoemulsification is the usual surgical treatment for a cataract that is impacting significantly on visual acuity [72]. Complete control of intraocular inflammation for at least 3 months prior to surgery is associated with improved outcomes [73, 74]. Aggressive courses of pre- and post-operative topical and systemic glucocorticoids may be required to optimise surgical results [40]. Surgical treatment may also be required for glaucoma associated with JIA-U unresponsive to pharmacological management. Interventional options include goniotomy, insertion of a glaucoma drainage device or trabeculectomy [75–77].

## Prognosis

Chronic anterior uveitis associated with JIA is a sight-threatening disease with ocular complications resulting from both the disease itself and its treatments. Visual loss may be present at first assessment with one study describing VA of 20/50 or worse in 40.3 % and 20/200 or worse in 24.2 % at presentation [78]. A systematic literature review looking at outcomes in JIA-U showed an adverse visual outcome (VA < 20/40 both eyes together) in 9.2 % of those with uveitis [3]. The main complications were cataracts, glaucoma and band keratopathy occurring in 20.5, 18.9 and 15.7 % respectively.

Several studies have looked at long-term follow-up. Skarin et al. reported a cohort of 55 JIA-U patients between 1973 and 1982 [79]. Seven years after uveitis onset, 42 % had cataracts and 5 % glaucoma. At 24 years, 51 % had cataracts, 22 % glaucoma and 49 % had signs of active uveitis or were receiving topical glucocorticoids for recent flares. Similar persistence into adulthood of asymptomatic uveitis in almost half of patients with JIA-U was seen in a cohort of 19 subjects who were born in 1976–1980 [80].

Several factors have been identified which are associated with a more severe course of uveitis and development of complications [81–83]. These risk factors for poor prognosis include: male gender; young age at onset of uveitis; short duration between onset of arthritis and development of uveitis; and presence of synechiae at first diagnosis of uveitis. A retrospective case series including 65 children with JIA-U showed significantly worse VA in boys versus girls at 1 year and 3 year follow-up [84]. Another study suggested that a shorter time interval between arthritis and uveitis onset is the main predictor of severity of uveitis [85]. Risk factors for visual loss, a key long-term outcome, were examined in a retrospective study with 596 affected eyes [78]. The overall incidence of visual loss to 20/50 or worse was 0.18/eye year (EY). The overall rate of developing a new ocular complication was 0.15/EY but significantly lower at 0.04/EY in those with no complications at baseline. The same study also showed bilateral uveitis, active uveitis (≥1+ AC cells or ≥ 0.5 vitreous haze), longer duration of uveitis, presence of posterior synechiae, abnormal intraocular pressure (IOP) and history of prior intraocular surgery were associated with worse vision during follow-up.

There are suggestions that both JIA-U prevalence and frequency of complications may be decreasing over time. A prospective, cross-sectional study from Germany including 18,555 JIA patients between 2002 and 2013 found a significant decrease in uveitis prevalence from 13.0 to 11.6 % [86]. There were also significant decreases in uveitis complications from 33.6 to 23.9 % (OR 0.94, *p* < 0.001). Over the same time period there were increases in use of synthetic DMARDs (39.8 to 47.2 %) and biologic DMARDs (3.3 to 21.8 %). Another study compared a cohort of patients with JIA-U from 1990–1993 (*n* = 239) with a 2000–2003 cohort (*n* = 240) and found a frequency of complications of 35 and 21 % respectively [87]. This apparent reduction may be related to earlier use of systemic immunosuppression, such as methotrexate, to treat joint disease. Evidence to support this comes from a subgroup (*n* = 3512) of the German 2002–2013 JIA cohort with disease duration < 12 months at first documentation and more than 2 years of follow-up [88]. Compared to those with no DMARD treatment in the year before uveitis onset, the risk of uveitis was significantly decreased by methotrexate (hazard ratio [HR] 0.63, *p* = 0.022), by TNF inhibitors (HR 0.56, *p* = 0.001) and by a combination of the two (HR 0.10, *p* = 0.001). The use of methotrexate early, specifically within the first year of JIA, was associated with a clearly reduced risk of uveitis compared with no early methotrexate (4.8 % vs 8.5 % respectively; HR 0.29, *p* < 0.001).

## Future directions

Better understanding of the pathogenesis of JIA-U may help to identify biomarkers, either genetic or perhaps plasma factors, which would allow stratification of patients to higher risk groups. These could be targeted with earlier and more aggressive therapy.

Key to improvement of therapies is the effective translation from bench to bedside [89]. High quality evidence for efficacy and safety of novel treatments specifically within a JIA-U population is required. The SYCAMORE study, a randomised placebo-controlled multicentre trial of adalimumab for JIA-U has completed recruitment and is now in follow-up [59, 90]. Another RCT of adalimumab is awaiting report from France [91]. The APTITUDE trial, an open-label study of subcutaneous tocilizumab for anti-TNF-refractory JIA-U is recruiting patients in the UK [92]. There are also smaller studies underway or planned examining tocilizumab and abatacept in JIA-U [93, 94].

## Conclusion

JIA-associated uveitis is the commonest extra-articular manifestation of JIA with significant numbers of children still developing sight-threatening complications. Close co-operation between paediatric rheumatologists and ophthalmologists and regular screening with slit lamp examination is essential for early diagnosis. Growing evidence supports the early use of systemic immunosuppression with the aim of quiescence of intraocular inflammation and avoidance of steroid-related side effects. A range of biologic drugs are being used, mostly on the basis of retrospective observational cohort studies, in methotrexate-resistant or intolerant cases. Prospective trials of adalimumab and tocilizumab which are now underway will provide further evidence about their efficacy and safety in treatment of JIA-U. A better understanding of the immunopathogenesis and identification of predictive biomarkers to target the widening therapeutic armamentarium will be a key goal in the years to come.

## Abbreviations

AC: anterior chamber
ANA: antinuclear antibody
DMARD: disease-modifying anti-rheumatic drug
EY: eye year
HLA: human leucocyte antigen
JIA: juvenile idiopathic arthritis
JIA-U: juvenile idiopathic arthritis-associated uveitis
MMF: mycophenolate mofetil
MTX: methotrexate
PY: patient year
RCT: randomised controlled trial
SUN: Standardisation of Uveitis Nomenclature
TNF: tumour necrosis factor
Treg: T regulatory cell
VA: visual acuity
